# Adaptive molecular convergence is pervasive across deep time and largely decoupled from phenotypic convergence

**DOI:** 10.1073/pnas.2616248123

**Published:** 2026-07-28

**Authors:** Cory A. Berger, Marina I. Stoilova, Rebecca M. Varney, Sam C. Abrams, Maria Pia Miglietta, Paulyn Cartwright, Todd H. Oakley

**Affiliations:** ^a^ Department of Ecology, Evolution, and Marine Biology, University of California, Santa Barbara, CA 93106; ^b^ Department of Ecology and Evolutionary Biology, University of Kansas, Lawrence, KS 66045; ^c^School of Biological Sciences, University of Nebraska, Lincoln, NE 68502; ^d^ College of Creative Studies, University of California, Santa Barbara, CA 93106; ^e^ Department of Marine Biology, Texas A&M University Galveston, Galveston, TX 77554

**Keywords:** convergent evolution, molecular evolution, phylogenomics, Medusozoa

## Abstract

Reuse of homologous genes during the evolution of similar traits or ecological transitions is often taken as evidence that evolution is repeatable at the molecular level. To study gene reuse, biologists frequently select specific convergent phenotypes and search for signatures of natural selection in genomes associated with those phenotypes. However, the causes and frequency of genome-scale molecular convergence remain unresolved, especially over deep timescales. We use phylotranscriptomics and analyses of sequence evolution to show that adaptive molecular convergence—defined as excess convergence of nonsynonymous substitutions between homologs, consistent with positive selection—is widespread across Medusozoa. Molecular convergence declines slightly over time but persists among lineages separated by over 600 My, consistently exceeding null expectations based on random overlap. Moreover, lineages sharing repeatedly evolved phenotypes (eyes, medusa loss, and upright colonies) do not exhibit elevated molecular convergence relative to other comparisons. Instead, convergence occurs idiosyncratically among species pairs and is broadly concentrated in genes associated with environment-facing functions, including metabolism, immunity, and xenobiotic processing. Our results suggest that selection often drives similar protein substitutions in disparate lineages, but that the selective causes of molecular convergence reflect multifaceted, lineage-specific interactions between organisms and their environments.

## Significance

Evolution frequently repeats, but how often similar traits arise from similar genetic changes remains unclear, especially over deep timescales. Here, we use the ancient and diverse lineage Medusozoa (jellyfish and relatives) to show that distantly related species often experience identical genetic changes driven by natural selection, even across more than 600 My of evolution. Surprisingly, this molecular convergence is not primarily associated with obvious, repeatedly evolved phenotypes. Instead, our results support a widespread background of adaptive molecular convergence that is mainly driven by diverse ecological pressures unique to specific lineages. This suggests high repeatability of molecular evolution, but the relationship between genetic and phenotypic convergence is complex.

A central question in biology is how often similar ecological challenges lead to repeated genetic changes, and how reliably those changes explain repeated phenotypic adaptations. Repeated origins of similar phenotypes across independent lineages suggest that natural selection drives evolution toward similar solutions under comparable ecological conditions. Furthermore, the use of homologous genes in convergent origins of similar traits or ecological contexts suggests that genetic paths may also be repeatable (1–4). However, establishing causal links between genes and phenotypes remains difficult because we lack functional information for most genes in most species (5). Consequently, biologists often rely on comparative genomic approaches that use signatures of selection to associate genes with repeated adaptations. These genome-wide approaches implicitly assume that the molecular convergence observed between phenotypically convergent taxa reflects the genetic basis of observed adaptations. However, adaptive molecular convergence may also reflect broader patterns of selection acting across many genes and ecological contexts, rather than the genetics of any particular trait (6). Distinguishing these possibilities requires understanding how frequently adaptive molecular convergence occurs across genomes and over deep evolutionary timescales, and whether it is systematically associated with particular phenotypic adaptations.

Patterns of molecular convergence across evolutionary time have already provided important perspectives, especially over relatively shallow timescales. An emerging pattern is a negative relationship between gene reuse and divergence time (2, 7), suggesting that molecular convergence between distant lineages may be rare. However, the magnitude and rate of this decline are poorly understood across deep timescales (>100 My), as we have few data concerning the frequency of molecular convergence between such distantly related species. It is also unclear whether this decline reflects decreasing reuse of genes underlying particular convergent adaptations or a broader decline in molecular convergence without direct relationships to phenotype and ecology. Such a decline may occur due to functional and structural divergence of genomes, including gene turnover and loss (2). It is challenging to draw general conclusions from the literature due to extensive heterogeneity in the datasets and statistical approaches used across studies and historical difficulties differentiating between adaptive and neutral molecular convergence (8, 9). In addition, many analyses focus on single-copy orthologs, which represent a small and potentially biased subset of genes (10, 11). Recent methodological advances help overcome these limitations. For example, CSUBST detects convergent amino acid substitutions consistent with positive selection across homologous proteins and can be applied to large multicopy gene families without focusing only on predefined convergent lineages (12). When combined with new phylogenetic methods for analyzing lineage-pair data—quantities defined for pairs of taxa such as convergence and divergence time (13)—these approaches allow genome-wide comparisons of adaptive molecular convergence across lineages and timescales within a unified statistical framework.

Here, we examine patterns of adaptive molecular convergence across Medusozoa (jellyfish and hydroids), an ancient and diverse clade in which key ecological phenotypes have evolved repeatedly over deep evolutionary time. These include multiple origins of eyes (14), repeated losses of the free-swimming medusa stage (15), and transitions in colony architecture (16). Because these convergent traits span hundreds of millions of years and diverse life histories, Medusozoa provides an unusually powerful system to test whether genome-wide molecular convergence predictably tracks repeated phenotypic adaptation over deep time. We quantify adaptive molecular convergence across thousands of gene families and compare its distribution across lineages differing in phenotype, divergence time, and ecological context. Our results reveal widespread molecular convergence that is not predictably associated with repeated phenotypic evolution, suggesting that protein sequence evolution can be strikingly repeatable even between distant relatives, while the selective causes of that repeatability are often diffuse, lineage-specific, and not easily identifiable from phenotype alone.

## Medusozoa Originated More than 650 Mya and Underwent Extensive Convergent Evolution of Phenotypes

**Fig. 1.**
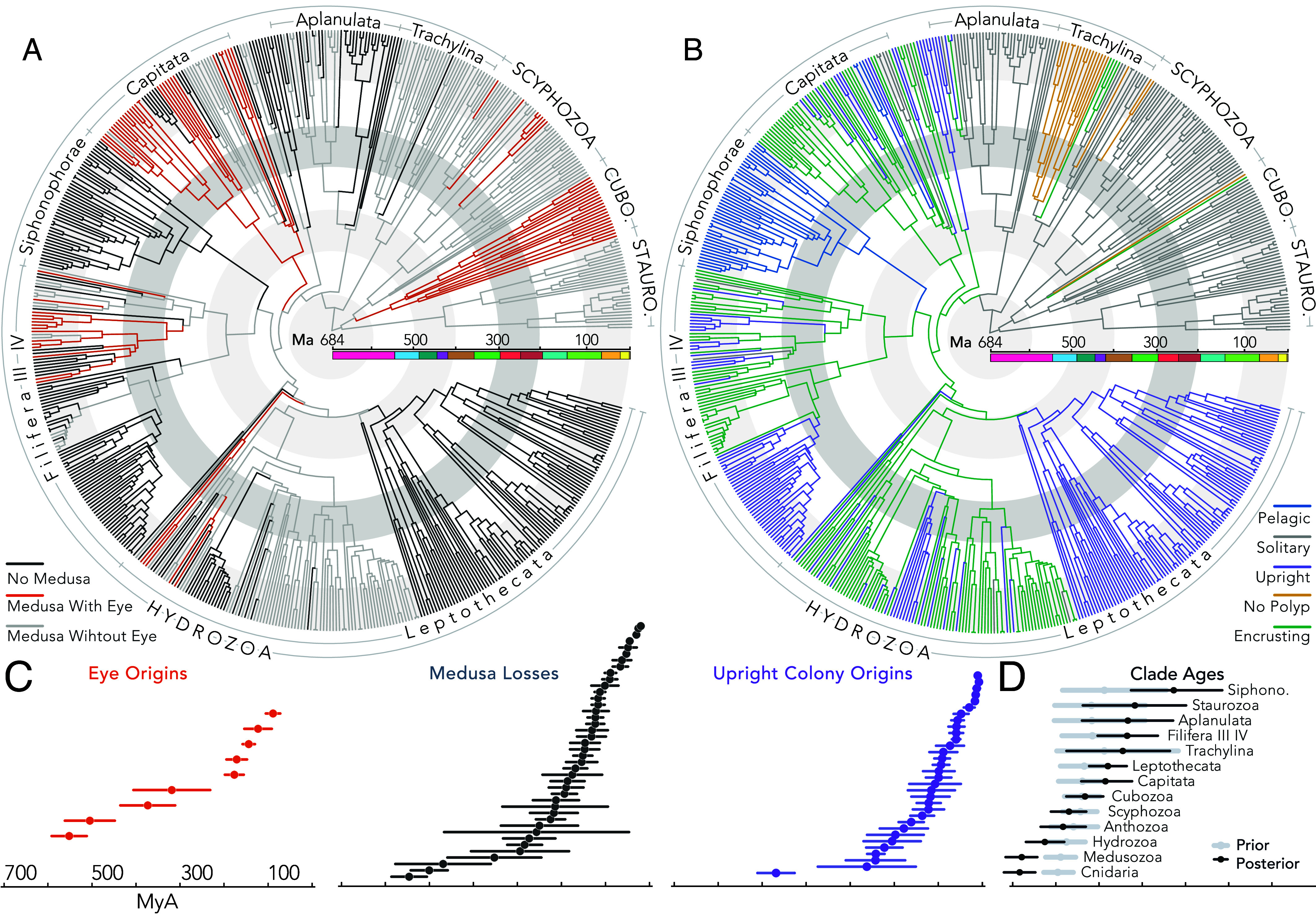
Convergent character transitions have occurred dozens of times across 680 My of medusozoan evolution. (*A*) Joint ancestral state reconstructions (ASR) of medusa stage and eye presence. ASRs were inferred using corHMM under the best-fit model by AIC (Dataset S4). Time-calibrated phylogeny was inferred from an MCMCtree analysis of 68 taxa chosen to evenly sample major clades; these times were transferred to a 561-taxon phylogeny for ASR. Filifera I-II are not highlighted here for space but can be found in the full trees on Dryad. “No medusa” includes medusoids, sporosacs, and absent gonophores. Medusa and eye presence were plotted together because these characters are correlated (eyes only occur on medusae; see *Materials and Methods*). Full species trees with taxon labels and branch support values can be found on the Dryad and Github; see *SI Appendix*, Fig. S17 for ASR of eyes only and *SI Appendix*, Fig. S18 for ASR of medusa stage with detailed character categories. (*B*) ASR of colony architecture. (*C*) Ages of key character transitions. Points and error bars show the mean and 95% CI of node ages calculated from 500 chronograms sampled from the MCMCtree posterior distribution. (*D*) Estimated ages of key clades. Points and error bars show the mean and 95% highest-posterior density intervals for the prior (fossil calibrations without sequence data) and posterior.

To better understand the timing and patterns of repeated evolution in Medusozoa, we used new and existing transcriptomic and genomic datasets for phylogenetic and character evolution analyses. Bayesian relaxed molecular clock analyses using 17 fossil calibrations (Dataset S3) firmly support a Cryogenian origin of Medusozoa (c. 685 Mya) and early diversification of major extant clades, including Ediacaran origins of Hydrozoa and Scyphozoa, an origin of Cubozoa near the early Cambrian, and Staurozoa later in the Paleozoic (Fig. 1). The resulting phylogeny resolves several previously uncertain relationships, most notably the placements of Capitata, Leptothecata, and the polyphyletic Filifera (Fig. 1 and *SI Appendix*, Figs. S10 and S11).

Using this time-calibrated phylogenetic framework, we reconstructed the evolutionary histories of multiple traits across Medusozoa, revealing extensive convergent evolution over deep time. At least nine independent origins of eyes, 41 losses of the medusa stage, and 32 transitions from encrusting to upright colony architectures were inferred (Fig. 1*C*). These repeatedly evolved traits are associated with distinct life histories and ecological strategies (15, 17, 18), providing a natural framework for testing links between phenotypic and molecular convergence. Across all three traits, the divergence times between lineages exhibiting convergent phenotypes spanned approximately 200 to 600 My, enabling comparisons between molecular and phenotypic convergence across deep evolutionary timescales.

## Adaptive Molecular Convergence Exceeds Null Expectations Across Deep Time

We quantified adaptive molecular convergence, defined as an excess of convergent nonsynonymous substitutions consistent with positive selection, across 86 medusozoan species using CSUBST (12). CSUBST identifies branch pairs in gene trees with an elevated rate of convergent nonsynonymous substitutions compared to the rate of convergent synonymous substitutions. This ratio, $\omega_{c}$, is analogous to but distinct from the well-known measure of sequence evolution ω; the neutral expectation of $\omega_{c}$ is 1, and $\omega_{c}\gg 1$ is evidence of convergent selection toward identical amino acids. For each pair of species, we calculated molecular convergence as the proportion of gene families with at least one convergent branch pair, not distinguishing between orthologs and paralogs (*Materials and Methods*).

**Fig. 2.**
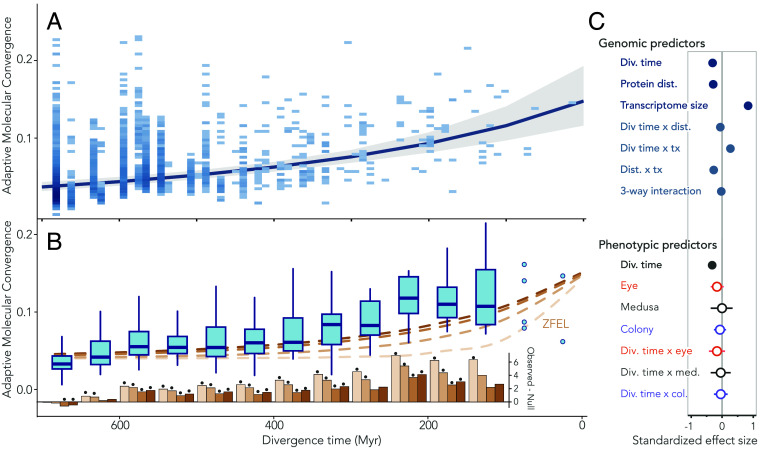
Adaptive molecular convergence declines with divergence time and exceeds null expectations. (*A*) Adaptive molecular convergence (AMC) among medusozoan lineages as a function of divergence time. AMC was quantified as the proportion of gene families exhibiting convergent branch pairs. AMC values were transformed with a Yeo-Johnson transformation (λ=−0.565); the regression is plotted here backtransformed onto the original scale. Points representing pairwise species comparisons (n=3,570) were binned for visualization purposes, with darker shading indicating higher observation density. The solid line shows the phylogenetic regression between AMC and divergence time, with the shaded ribbon indicating the model CI. (*B*) Observed AMC compared with expectations under Zero-Force Evolutionary Law (ZFEL) simulations. Boxplots show the distribution of observed convergence (data in Panel *A*) binned into 50 My intervals. Individual points were plotted for intervals with <10 data points. Dashed lines show median convergence expected from ZFEL simulations, which model the decline in similarity among independently evolving systems without correlated selection. The multiple lines show different realizations of the null with varying rates of loss. All scenarios are set to converge on the same steady-state value, which equals the convergence in the final time point of observed data (∼4%; see *Materials and Methods*). Below, bars show mean differences between observed and null values within each bin. Asterisks indicate P<0.05 (Bonferroni-corrected within each null model). The most conservative null distribution (dark brown) was constructed such that its lower 0.025 quantile would include the observed value at our oldest time point; this explains why null values slightly exceed observed values in the oldest bin (*SI Appendix*, Fig. S15 and *Supplemental Text*). (*C*) Predictors of adaptive molecular convergence. *Top*, standardized regression coefficients from a model evaluating the effects of divergence time, protein sequence distance, transcriptome size, and their interactions on AMC. CIs are smaller than points. *Bottom*, standardized regression coefficients for a model testing whether eyes, medusa stage, and colony form predict AMC. Horizontal bars indicate CIs and shaded circles indicate significance (P<0.05).

Adaptive molecular convergence occurred broadly across medusozoan lineages and genes. In total, we identified 426,322 convergent branch pairs (0.091% of all tested branch pairs) across 8,991 of 9,194 gene families. Phylogenetic regression showed that the degree of molecular convergence declined significantly with increasing divergence time (Fig. 2*A*; P≪0.01), consistent with predictions that distantly related taxa have fewer opportunities for shared substitutions (2). Nonetheless, there was substantial variation across all divergence times, indicating that phylogenetic relatedness and evolutionary time explained only a small fraction of the extensive molecular convergence in our data [residual SE (RSE) = 0.82 on the transformed scale]. Sequence data simulated from our alignments under a scenario of zero convergence resulted in a low false positive rate and no relationship with divergence time (*SI Appendix*, Fig. S6 and *Supplemental Text*), indicating that observed patterns of convergence cannot be attributed to biases in the underlying alignments and gene trees. Furthermore, convergent substitutions identified by CSUBST were less physicochemically conservative than other substitutions in the same proteins (*SI Appendix*, Fig. S7). This pattern was not observed in the data simulated without convergent selection, bolstering inferences that most convergence events identified by CSUBST were driven by positive selection.

Do levels of adaptive molecular convergence across Medusozoa reflect systematic constraints and selection, or can they simply arise from random overlaps among loci in finite genomes? To test this, we simulated null expectations of convergence derived from the Zero-Force Evolutionary Law (19), which states that similarity among independently evolving systems declines over time in the absence of constraints or directional forces, such as selection acting the same way in both systems. Accordingly, if molecular convergence reflects only uncorrelated selection across genes, it will decline with increasing divergence time and not exceed the levels expected from random overlap alone (*Materials and Methods*).

Levels of adaptive molecular convergence in Medusozoa consistently exceeded these null expectations, even across deep evolutionary time. Across a 650 My range of divergence, molecular convergence was significantly higher than predicted under random overlap alone (Fig. 2*B*). Null models were constructed under a range of possible parameter values (*Materials and Methods*), and convergence exceeded null predictions across most time intervals even under the most conservative conditions (dark brown in Fig. 2*B*), which correspond to the slowest possible decline compatible with uncorrelated evolution. The persistent excess of observed convergence under these very conservative conditions indicates that adaptive molecular convergence in Medusozoa reflects systematic evolutionary forces and/or constraints operating across deep time.

## Adaptive Molecular Convergence Is Not Explained by Phenotypic Convergence

If adaptive molecular convergence primarily reflects reuse of genes underlying particular convergent traits, then species with those traits should show higher levels of molecular convergence. To test this prediction, we compared levels of molecular convergence among species pairs with phenotypic convergence of eyes, upright colony architectures, or losses of the medusa stage to those of other species pairs without obvious convergent traits. Contrary to expectations, species with convergent phenotypes were statistically indistinguishable from other taxa in both overall levels of molecular convergence (P>0.1) and the effect of divergence time (P>0.1) (Fig. 2*C*). Therefore, these three prominent, ecologically important, and repeatedly evolved phenotypes do not explain the observed genome-wide patterns of adaptive molecular convergence.

We next asked whether other biological factors could account for variation in convergence among species pairs. Incorporating pairwise protein distance and transcriptome size as covariates in addition to divergence time improved regression model fit, and all main effects and interactions were significant (Fig. 2*C* and *SI Appendix*, Table S5). As expected, species with greater protein sequence similarity exhibited higher overall levels of molecular convergence and maintained elevated convergence over longer evolutionary intervals. The same was true for species with larger transcriptomes, likely because additional gene copies provide more opportunities for convergence within gene families (*SI Appendix*, Fig. S8). This suggests that gene duplication may be an important contributor to patterns of molecular convergence by providing evolutionary substrates for convergent selection, while loss and divergence of orthologs generally reduces these opportunities over time (*SI Appendix*, Fig. S8). Nevertheless, substantial unexplained variation remained even after accounting for these covariates (RSE = 0.77). We further tested whether adaptive molecular convergence was systematically associated with particular clades or taxonomic groups. After accounting for transcriptome size, species-averaged convergence values lacked detectable phylogenetic signal (P=0.8), and taxa with high or low convergence showed no obvious shared characteristics (*SI Appendix*, Fig. S9).

Together, these results indicate that adaptive molecular convergence varies widely among species pairs and cannot be readily attributed to phenotypic similarity or phylogenetic structure. Consistent with this decoupling, although genes with established roles in eye development were moderately enriched in comparisons between eye-bearing species, these genes also often experienced convergent selection in species lacking eyes (*SI Appendix*, Fig. S16 and Table S2). Thus, genomic scans may enrich for trait-related genes, but the pervasive background of adaptive molecular convergence makes it difficult to interpret apparent gene reuse as evidence of trait-specific adaptation.

## Adaptive Molecular Convergence Reflects Multifaceted Organism–Environment Interactions

Convergence may exceed null expectations and be unequally distributed across gene families due to either shared genomic constraints [e.g., similar mutational landscapes, genome architectures, and gene-regulatory networks; (1, 20)] or shared ecological contexts resulting in similar adaptive landscapes (21). We can partially disentangle these factors because genomic constraints should decline with phylogenetic distance (22–25), whereas ecological distance may lack phylogenetic signal (26, 27), particularly across divergent clades. We therefore predict that, if convergence is primarily structured by genomic constraints, then closely related taxa will share more similar sets of convergent genes.

**Fig. 3.**
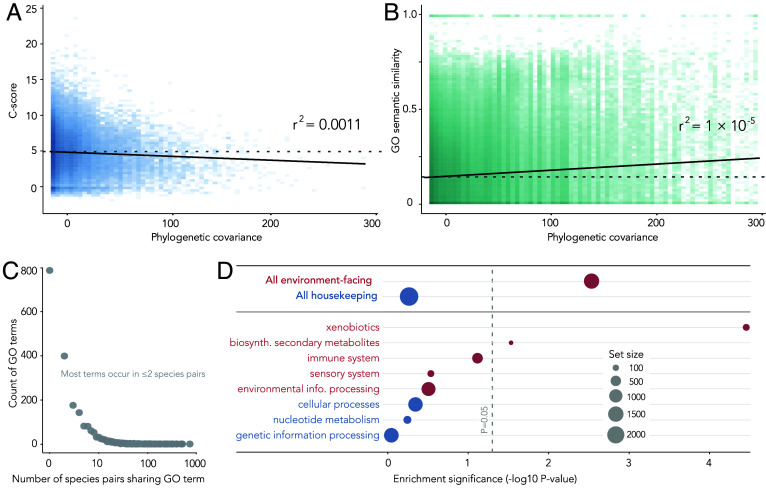
Adaptive molecular convergence lacks phylogenetic structure but is associated with environment-facing genes. (*A*) Identities of convergent genes are not associated with phylogenetic distance. C-scores represent the overlap of gene families that independently experienced convergence in two pairs of species, with each unit increase representing one SD above the expected overlap from a null hypergeometric distribution. Each point shows the C-score of a randomly chosen species quartet (n=236,070) plotted against the phylogenetic covariance of that quartet, with darker shading indicating higher observation density. Higher covariance values indicate more shared phylogenetic history. The dashed line indicates average value and the solid line indicates a linear regression. (*B*) GO annotations of convergent genes are not associated with phylogenetic distance. Each point represents the mean semantic similarity of the lists of GO terms enriched among convergent genes between two pairs of species (n=4,755,188 quartets) plotted against phylogenetic covariance. Semantic similarities were calculated as the mean of the maximum similarity between each GO term and members of the other list. The dashed line indicates average value and the solid line indicates a linear regression. (*C*) Number of species pairs with enrichment of each GO term (log scale on X axis). Most GO terms are only found in one or two species pairs. (*D*) Gene set enrichment analysis of “environment-facing” and “housekeeping” genes. Genes were categorized based on KEGG annotations and grouped into the subcategories shown here and in *SI Appendix*, Table S4. The dashed line indicates raw *P*-value = 0.05, and point sizes indicate the number of annotated genes in that category.

To test this, we quantified the frequency with which members of the same gene family independently experienced convergent selection across different species pairs using an extension of Yeaman et al.’s C-score metric (28). Convergent gene families were similar across species quartets (pairs of pairs) more frequently than expected under random chance (mean C-score = 4.91±2.72), with 33.9% of quartets showing significant overlap (Bonferroni-corrected P<0.01). However, similarity in the identities of convergently evolved genes showed no meaningful relationship with phylogenetic distance ($r^{2}=0.0011$; Fig. 3*A*). Thus, although adaptive molecular convergence is repeatable at the gene-family level, it is not preferentially shared among closely related taxa, arguing against a dominant role for shared genomic constraints in structuring molecular convergence across Medusozoa. Patterns of molecular convergence were similarly unstructured at the level of gene function. Gene ontology (GO) terms enriched among convergent genes were unevenly distributed across species pairs, with most terms restricted to one or two comparisons (Gini coefficient = 0.78; Fig. 3*C*). Semantic similarity of enriched GO terms among species quartets showed no relationship with phylogenetic distance ($r^{2}=1\times 10^{-5}$, Fig. 3*B*), indicating that convergently adapted functions are largely lineage-specific and lack phylogenetic structure. These results further suggest that genomic constraints alone cannot explain which genes or functions repeatedly experience convergent selection.

Despite this lack of phylogenetic structure, adaptive molecular convergence was strongly nonrandom with respect to gene function across Medusozoa. Gene families with high convergence rates were enriched for functions related to energy metabolism, immune response, and xenobiotic processing, whereas genes with low convergence rates were enriched for internal regulatory processes, including gene regulation, meiosis, and the cell cycle (*SI Appendix*, Fig. S10). Using an alternative classification based on KEGG pathway annotations, we grouped genes a priori into environment-facing and housekeeping categories and tested for differences in convergence between these classes (*SI Appendix*, Table S4). Environment-facing genes were significantly enriched among convergent genes (q=0.00308; Fig. 3*D*), whereas housekeeping genes were not (q=0.286). Together, these results indicate that adaptive molecular convergence is concentrated in genes involved in diverse organism–environment interactions rather than internal housekeeping functions, consistent with selection associated with multiple ecological axes across deep evolutionary time.

## Discussion

We find adaptive molecular convergence to be pervasive across medusozoan evolution over deep time. However, this convergence is not predictably associated with phenotypic or phylogenetic similarity, and instead reflects broader, lineage-specific ecological pressures. Our results suggest that adaptive molecular convergence is widespread but usually difficult to relate to specific phenotypes or selective agents. Here, we discuss the implications of this pattern for three related questions: The drivers of molecular convergence over deep time, the interpretation of genomic signatures of adaptation, and the predictability of evolutionary outcomes.

First, we argue that molecular convergence over deep time is not primarily structured by shared genomic constraints, but instead reflects selection across complex ecological dimensions. The identities and functions of convergent genes are not associated with phylogenetic distance in Medusozoa, indicating that constraints, which should decay with genetic divergence (22–25), do not determine which genes repeatedly experience convergent selection. We do not exclude that constraints influence molecular convergence (1, 29), but our data suggest that genome-wide patterns of adaptive convergence are mainly driven by selection on diverse, often unobserved, ecological factors. As a result, genes that interface with the external environment show elevated rates of convergence overall, and individual events of molecular convergence occur idiosyncratically across lineages. Higher rates of convergence in environment-facing genes could also be caused by higher rates of protein evolution, which vary across gene functional categories (e.g., ref. 30). Nonetheless, this pattern is consistent with observations that adaptive convergence occurs across genes with diverse functions (31) and reflects the idea that selective regimes are highly multidimensional (6, 32). Species therefore experience many uncorrelated and lineage-specific selective pressures that may overlap among taxa, such that the underlying selective agents are often unknown.

Second, this perspective reshapes interpretations of genome-wide molecular convergence and helps reconcile seemingly incongruent patterns in the literature. On the one hand, many studies have identified repeated use of specific genes or pathways associated with convergent phenotypes or ecological transitions (2, 11). On the other hand, genome-wide analyses have often failed to detect elevated molecular convergence in lineages with repeatedly evolved traits (33–38), a pattern frequently attributed to a pervasive background of neutral convergence (4, 39); but see refs. 35 and 36. However, we have lacked estimates of the distribution of adaptive convergence across taxa and whether it exceeds expectations under independent evolution. We show that, in addition to a neutral background (9), there is also a widespread background of adaptive convergence that is not restricted to specific traits. As a result, molecular convergence is not necessarily enriched in focal lineages, even when phenotypes have evolved repeatedly, because trait-specific signals are diluted within this adaptive background. This perspective also reframes patterns such as the reported decline in gene reuse over time (2). Although the decline is real, it may not reflect decreasing reuse of genes underlying specific adaptations. Our results suggest that a similar pattern arises even from comparisons among randomly chosen taxa. An important direction for future studies will be to explicitly quantify axes of ecological variation, which could account for some of the variables driving convergent selection (21, 32).

We used amino acid substitutions as a signature of convergent selection because this approach is uniquely tractable across long timescales and large gene families. However, convergent substitutions represent a limited subset of evolutionary change and do not correspond to functional units as clearly as coding regions or regulatory elements, which often experience convergent selection via nonidentical substitutions. Further work is needed to understand patterns of convergence across time at other genetic and molecular levels, which can be uncoupled from one another (40). Regulatory evolution, for instance, may be more important than protein sequence changes for many convergent phenotypes, particularly morphological traits (41, 42). Therefore, integrating multiple signatures of convergence across coding sequences, regulatory regions, and gene expression may provide a more complete view of the genomic basis of repeated adaptation (43). Such analyses remain limited in taxonomic and temporal scope by the availability of high-quality genomes, and further theoretical advances are also needed to better associate regulatory and expression changes with positive selection (e.g., ref. 44).

Finally, our results reveal a tension between two perspectives on evolutionary repeatability. The pervasive nature of adaptive molecular convergence indicates that protein evolution is unexpectedly repeatable even among highly divergent lineages. At the same time, the lack of consistent mapping between molecular convergence and phenotype suggests that this repeatability is not easily interpretable or predictive. This is highlighted by the observation that homologs of known eye-related genes consistently experienced convergent selection in species without eyes. More generally, genomic signatures of selection do not directly identify the ecological causes of selection. At the amino acid level, processes such as pleiotropy, epistasis, and functional divergence can produce convergent substitutions that do not confer convergent functions. Similar principles apply at other levels of organization, including morphology: Similar phenotypes can arise from different selective pressures and similar selective pressures can produce different phenotypes (40).

Despite these challenges, many studies have convincingly linked convergent molecular changes with adaptive phenotypes, including convergent substitutions in digestive proteins of carnivorous plants (45) and in proteins expressed in the inner ears of echolocating mammals (46, 47). A particularly compelling example is the repeated evolution of resistance to toxic cardiac glycosides via convergent substitutions in a sodium–potassium pump, which has occurred in amphibians, reptiles, mammals, and insects (48). Both plants and toads produce these toxins, so distantly related species that consume either of these prey items face the same selective pressure and have evolved similar molecular solutions. This illustrates one way that specific selective forces can drive molecular convergence in otherwise dissimilar taxa. Although establishing these genotype–phenotype relationships requires multiple lines of evidence and, ideally, labor-intensive functional characterization, molecular convergence provides important clues about the ecological factors shaping genome and phenome evolution. Our finding of widespread adaptive convergence at the amino acid level suggests that many such examples await discovery.

## Materials and Methods

### Assembly Construction and Phylogenetics.

We assembled a dataset of 86 medusozoan genomes and transcriptomes and 12 anthozoan outgroups, including 22 transcriptomes newly generated for this study (Dataset S1). We used a common assembly and filtering pipeline for public and newly assembled transcriptomes and a separate pipeline to infer protein-coding genes from genomes (*SI Appendix*, *Materials and Methods*). We inferred orthogroups (OGs) using an iterative approach to minimize errors from contamination, homology inference, and sequencing artifacts. Briefly, we used OrthoFinder v2.5.5 (49) with the settings “-y -M msa” to obtain Orthogroups (OGs) present in at least 30/98 species. For each OG, we masked nonhomologous characters using PREQUAL (50), aligned sequences using MAFFT (51), trimmed sites with <10% occupancy using TrimAl (52), and reinferred gene trees using either 1) IQ-Tree v3.0.1 (53) with settings “-fast -mset LG+G -m TEST -alrt 1000” (for OGs with ≤1,000 sequences); or 2) FastTree with settings “-LG -gamma -pseudo” (for OGs with >1,000 sequences). We then used TreeShrink v1.3.9 (54) with alpha=0.10 in ‘all-genes’ mode to remove potential erroneous sequences on long branches. Next, we realigned and trimmed cleaned OGs, reinferred trees using IQ-Tree (-fast -m Q.pfam+F+R10), reran TreeShrink, and inferred final gene trees using IQ-Tree with the model Q.pfam+F+R10.

We extracted single-copy orthologs using the Maximum Inclusion algorithm of PhyloPYPrunerhttps://pypi.org/project/phylopypruner/. These orthologs were filtered and aligned as above, and we built final single-copy gene trees using IQ-Tree with the best-fitting model (-msub nuclear). We obtained 1771 orthologs present in at least 30 species. Finally, we excluded 29 genes (1.6%) that failed IQ-Tree’s symmetry tests (P<0.05) and proceeded to analyze the remaining 1742 loci.

We built maximum-likelihood (ML) gene trees with IQ-Tree with best-fit models selected by ModelFinder, and inferred species trees using ASTRAL (55) and IQ-Tree. We also inferred a coalescent tree from 9194 multicopy gene families using ASTRAL-PRO3 (55). These approaches recovered the same relationships among major clades with strong support (*SI Appendix*, Figs. S10 and S11). Some shallow relationships were unresolved in the IQ-Tree and ASTRAL trees because these taxa contained little overlap in our data matrix (*SI Appendix*, Table S1), but these relationships were successfully resolved with ASTRAL-PRO3 (55). We used the well-resolved phylogenomic topology as a backbone for a 621-taxon phylogeny (563 medusozoans and 58 anthozoans) derived from DNA barcoding sequences (14). We combined this DNA data matrix in a partitioned analysis together with 100 amino acid alignments in IQ-Tree (*SI Appendix*, *Materials and Methods*), constrained to match the consensus topology from the above species trees.

### Divergence Dating and Fossil Calibrations.

We selected 50 genes for divergence dating using kinda-date (56) and performed relaxed molecular clock analyses using the independent-rates model of MCMCTree (57). We included a series of sensitivity analyses considering variation in rate priors, gene sampling, and taxon sampling (*SI Appendix*). To run MCMCTree, we used the coalescent species topology and estimated Hessian matrices and branch lengths under the LG substitution matrix and a five-category gamma model of rate variation. We ran each MCMCTree analysis with two chains, sampling every 500 iterations for 20,000 samples following a burn-in of 1,000,000 iterations, and also performed MCMC sampling from the prior using the same settings. Fossil constraints are described in Dataset S3. All constraints are truncated Cauchy distributions with 2.5% soft minima unless otherwise noted. We constrained the root using a skew-normal distribution with a hard minimum and a 5% soft maximum such that the 0.95 quantile was 609 Mya [the age of the Lantian biota (58)].

Finally, we transferred divergence times from MCMCTree to the 621-taxon phylogeny using Congruify (59) and TreePL (60) (with the smoothing parameter $1\times 10^{-11}$. We did this for the mean posterior MCMCTree as well as for 500 trees sampled from the posterior to account for divergence time uncertainty.

### Ancestral State Reconstructions.

We conducted literature searches for eye presence/absence, medusa/gonophore morphology, and colony architecture (Dataset S4). For eyes, we followed (14) in defining eyes minimally as a “region made of photoreceptor cells adjacent to pigment cells.” For medusae, we used an encoding scheme to capture the continuum of reduced medusa morphologies based on Cartwright and Nawrocki (16) and Miglietta and Cunningham (15): 1) motile, autonomous feeding medusae, 2) nonfeeding medusoids with radial canals and/or tentacle bulbs, 3) gonophores lacking radial canals and tentacle bulbs (sporosacs), and 4) gonophores absent. To provide realistic models of character evolution, we disallowed regains of medusae from any other state (15). For colony architecture, species were scored as: 1) lacking a polyp stage, 2) solitary, 3) encrusting colonies, 4) upright colonies, and 5) pelagic colonies. We did not allow polyps to be regained once lost. Additionally, based on initial analyses, we found that upright and pelagic colonies were never lost and therefore also disallowed losses of those states.

We inferred ancestral state reconstructions (ASRs) using corHMM v2.10 (61) with the “maddfitz” root prior and 10 random restart, tested models with up to 4 hidden rate categories, and reported the best-fitting models in the main text (Dataset S4). We inferred ASRs on a phylogeny without Anthozoa because anthozoan colonies are not homologous to medusozoan colonies (62) and our encoding scheme does not apply to them. We identified conservative minimum numbers of character transitions by manually inspecting node marginal likelihoods and uncertainty (bootstrap support) in the species tree. Specifically, we considered nodes with bootstrap support <80% as unsupported; if an ASR inferred multiple origins separated by such an unresolved node, we conservatively consider this a single origin. To estimate ages of character transitions, we examined the posterior distributions of relevant node ages from 500 randomly sampled posterior trees. We also tested for phylogenetic correlations between characters using the test proposed by Boyko and Beaulieu (63) (*SI Appendix*, *Materials and Methods*).

### Analyzing Adaptive Molecular Convergence.

We used CSUBST (12) to infer convergence events between each pair of species (n=86 medusozoans, n=3,570 species pairs) using the set of 9194 OGs used for the ASTRAL-PRO analysis. Gene trees were midpoint rooted and codon sequences were obtained using Pal2nal v14.1 (64). After running CSUBST in “analyze” mode on each gene tree and codon alignment with setting “–max_arity 2,” we extracted all branch pairs with at least 3 nonsynonymous convergent substitutions (OCNany2spe≥3) and omegaCany2spe≥3, as recommended by the CSUBST authors, and considered these branch pairs “convergent.” To subset data for the eye, medusa loss, and coloniality data, we extracted only the comparisons between phenotypically convergent lineages identified by ASR. For medusa-loss species, we further excluded medusoids and only considered comparisons between species with either sporosacs or no gonophores. For colonial species, we considered comparisons between species with upright colonies. See *SI Appendix*, *Materials and Methods* for details and discussion of technical considerations for scoring convergence.

For each pair of species, we calculated the proportion of OGs with at least one convergence event between them and performed phylogenetic regression analyses to test for a relationship with divergence time. We accounted for phylogenetic structure by calculating the phylogenetic covariance matrix for species pairs with the phylopairs R package of Anderson et al. (13). We used this covariance matrix as input for phylogenetic generalized least-squares (PGLS) analyses with the “gls” function of the nlme R package, and applied a Yeo-Johnson transformation (65) on convergence values because they were right-skewed (*SI Appendix*, Fig. S13). In addition to divergence time, we considered median pairwise protein distance and mean transcriptome size as covariates. To test for differences between eye, medusa loss, and coloniality data subsets, we coded each group as a binary variable and tested for group differences and interaction terms. See *SI Appendix*, *Materials and Methods* for further details on the above analyses.

### Null Model of Convergence.

We used two-state Markov chains to model the expected decline of convergence over time in the absence of systematic forces or constraints. See *SI Appendix* for further discussion. We modeled genetic loci (here, gene families) as binary variables that can transition from 0 to 1 with probability $P_{\mathrm{gain}}$ or from 1 to 0 with probability $P_{\mathrm{loss}}$. A locus is convergent if it is 1 (i.e., “selected”) in both species. This is a two-state Markov chain with steady-state frequency of

[1] $$\begin{array}{c} S=P_{\mathrm{gain}}/\left(P_{\mathrm{loss}}+P_{\mathrm{gain}}\right) \end{array}$$

Ref. 66. Given two vectors of n loci, the expected proportion of overlap due to random chance is simply the product of the proportions of ones in each vector, which at equilibrium equals $S^{2}$. Given $P_{\mathrm{loss}}$, $P_{\mathrm{gain}}$, and a starting proportion of ones $S_{0}$, we can use random walks to simulate convergence over time, which will eventually reach the steady state. We estimated $S_{0}$ as the square root of the fitted divergence time regression at time 0 ($S_{0}=0.3878$). This is the number of “selected” loci that must exist on average to explain the observed level of convergence, assuming all overlap is simply due to chance. We then assumed that S is the square root of the mean value at the oldest time point in our dataset, 681.94 Mya (S=0.201).

### C-Scores and Gene Enrichment Analyses.

We calculated the overlap of convergent genes for pairs of species pairs (quartets). Specifically, we calculated the ratio of OGs that independently experienced convergence in both pairs to the set of all OGs found in both species pairs, and calculated C-scores from the hypergeometric distribution (28). To ensure that we only considered independent convergence events, we only counted convergence events that occurred along different branches of a gene tree. This procedure is computationally intensive because we have to map substitutions onto gene trees. Rather than analyze all 4,755,188 possible nonoverlapping quartets, we drew 50 random samples of 100 species pairs each, resulting in 247,500 total quartets (236,070 after excluding species overlaps). We then used the covariance between species pairs in the lineage-pair phylogenetic covariance matrix as a measure of phylogenetic distance and performed a linear regression between C-scores and covariance using R’s “lm” function.

We performed GO enrichment analyses of convergent genes for each species pair using TopGO (67) with the Fisher’s exact test and “weight01” algorithm, and considered GO terms significant at a level of P<0.001. We calculated the semantic similarity between all combinations of the 2082 unique significant GO terms using REVIGO (68). We then compared lists of GO terms for all 4,755,188 species quartets using the “Best Match Average” method (69). We regressed these values against phylogenetic covariance using “lm,” as above. We also performed GO enrichment at the OG level across all species using the “KS test” functionality of TopGO with the per-gene convergence rate as input.

To test whether genes with known roles in phototransduction and cnidarian eye development experienced higher rates of convergence among eye-bearing species, we compiled two sets of genes: 1) homologs of differentially expressed genes upregulated in the eye-bearing tissues of *Aurelia aurita*, *Tripedalia cystophora*, and *Stachys tubulosa* (70), and 2) gene families belonging to a previously defined list of light-interacting genes (71). We added a handful of genes known to play roles related to light perception in cnidarians [Pax transcription factors and a hydrozoan eye opsin; (70)] and modified the set of circadian-related genes to reflect current knowledge of clock genes in Medusozoa (72). In total, we identified 798 eye-associated OGs belonging to these gene sets.

We used KEGG pathway annotations to classify certain gene sets as either “environment-facing” or “housekeeping.” This allows us to test whether these a priori-defined gene sets differ in their levels of convergence, orthogonal to the above exploratory analyses based on GO terms. Specifically, we retrieved OGs annotated with KEGG terms we considered to fall unambiguously into these two categories (*SI Appendix*, Table S4). We then ranked OGs according to their per-gene convergence rate as above and used the “GSEA” function of the clusterProfiler R package to test for enrichment of these two gene sets. For visualization purposes, we also calculated enrichment of the individual KEGG categories (Fig. 3*D*).

## Supplementary Material

Appendix 01 (PDF)

Dataset S01 (XLSX)

Dataset S02 (CSV)

Dataset S03 (PDF)

Dataset S04 (XLSX)

Dataset S05 (XLSX)

## Data Availability

Sequence data generated for this study are available at NCBI under Bioproject PRJNA1455705, and accessions of previously published data can be found in Dataset S1. Transcriptome assemblies, alignments, gene and species trees, CSUBST output, and other large data files are available on Dryad (73). Scripts and smaller data files are available at https://github.com/ucsb-oakley-lab/Medusozoa_project (74) and will are also archived on Dryad. All other data are included in the manuscript and/or supporting information.
