# Supplementary material for: Adaptive molecular convergence is pervasive across deep time and largely decoupled from phenotypic convergence
Source: Proc Natl Acad Sci U S A. 2026 Jul 28;123(31):e2616248123. doi: 10.1073/pnas.2616248123 (PMC13438476; doi:10.1073/pnas.2616248123)
Supplement: Supplementary file 2 [file pnas.2616248123.sd03.pdf]

Fossil calibrations used in Berger et al.

Node: Cnidaria
Calibration: Skew-normal distribution with 563.7 Mya hard minimum and 609 Mya 5% soft maximum ('SN(5.637, 0.2312, 183.45)').
Minimum Age: 563.7 Mya
Fossil & Justification: *Haootia quadriformis* from the lower Fermeuse Formation, dated to between 563.67–569.01 Mya (Matthews et al., 2021). Has been interpreted as a stem staurozoan, but this is uncertain (Van Iten et al., 2016). We conservatively use this fossil to constrain crown Cnidaria.
Maximum Age: 609 Mya
Justification: Age of the Lantian biota, recently dated to 602 +- 7 Mya (Yang et al., 2022). This formation preserves many exceptional algal macrofossils but no definitive crown metazoans.

Node: Acraspeda
Calibration: 'L(5.419, .1, 0.25, 0.025)'
Min Age: 541.9 Mya
Fossil & Justification: *Paraconularia ediacara* from the Ediacaran Tamengo Formation (Leme et al. 2022), dated to 542.27 ± 0.38 Mya (Parry et al., 2017). Conulariids may have affinity to scyphozoans or staurozoans, but in any case can reliably constrain crown Acraspeda.

Node: Carybdeida
Calibration: 'L(5.05, .1, 0.25, 0.025)'
Min Age: 505 Mya
Fossil & Justification: Specimens UU07021.01 and UU07021.02 from Cartwright et al., 2007. Cambrian Series 3 Marjum Formation (Utah, USA; c. 505 Ma) yields fossils identified as crown cubozoans with likely affinity for Carybdeida (Cartwright et al., 2007; Van Iten et al., 2014).

Node: Chirodropidae
Calibration: 'L(3.09, .1, 0.25, 0.025)'
Min Age: 309 Mya
Fossil & Justification: *Anthracomedusa turnbulli,* Upper Carboniferous, Mazon Creek Formation (Young & Hagadorn 2010).

Node: Scyphozoa+Cubozoa
Calibration: 'L(5.145, .1, 0.25, 0.025)'
Min Age: 514.5 Mya
Fossil & Justification: *Yunnanoascus haikouensis* from the Lower Cambrian Heilinpu Formation (Han et al., 2016), Cambrian Stage 3 (514.5 - 521 Ma). Explicit phylogenetic analyses place as a stem scyphozoan, so we use this fossil to constrain the split between Scyphozoa and Cubozoa.

Node: Rhizostomeae
Calibration: 'L(3.05, .1, 0.25, 0.025)'

Min Age: 305 Mya
Fossil & Justification: *Prothysanostoma eleanorae*, Upper Carboniferous; Cherryvale Formation/Wea Shale, IA. Missourian Stage (Young & Hagadorn 2010).

Node: Coronatae
Calibration: 'L(5.05, .1, 0.25, 0.025)'
Min Age: 505 Mya
Fossil & Justification: Specimen UU07021.05 from Cartwright et al., 2007. Cambrian Series 3 Marjum Formation (Utah, USA; c. 505 Ma) yields fossils identified as crown scyphozoans with likely affinity for Coronatae (Cartwright et al., 2007; Van Iten et al., 2014).

Node: Hydrozoa
Calibration: 'L(5.05, .1, 0.25, 0.025)'
Min Age: 505 Mya
Fossil & Justification: Cartwright et al, 2007; Van Iten et al., 2014: "The oldest known hydrozoans and cubozoans are represented by medusae in the Cambrian (Series 3) Marjum Formation (Utah, USA; *c*. 505 Ma)"

Node: Limnomedusae
Calibration: 'L(2.42, .1, 1, 0.025)'
Min Age: 242 Mya
Fossil & Justification: *Progonionemus vogesiacus*, Lower Triassic; Grès à Voltzia Formation, Middle Triassic (Young & Hagadorn 2010).

Node: Plumularioidea
Calibration: 'L(3.827, .1, 0.25, 0.025)'
Min Age: 382.7 Mya
Fossil & Justification: *Archaeoantennularia byersi* Decker, 1952 and *Plumalina densa* Hall, 1878. Middle Devonian, Sylvania, Michigan, USA and Chemung, Belvidere, New York, USA (Song et al., 2021).

Node: Hydractiniidae
Calibration: 'L(0.66, .1, 1, 0.025)'
Min Age: 66 Mya
Fossil & Justification: *Psammoactinia antarctica* Olivero and Aguirre-Urreta, 1994. Late Cretaceous, Early Maastrichtian, Sanctuary Cliffs, Snow Hill Island, Antarctica (Song et al., 2021).

Node: Porpitidae (stem)
Calibration: 'L(4.211, .1, 0.25, 0.025)'
Min Age: 421.1 Mya
Fossil & Justification: *Pseudodiscophyllum windermerensis*. Late Silurian, Ludlow, Ludfordian, Bannisdale Formation, England (Fryer and Stanley, 2004).

Node: Filifera 1+2 (Proboscidactyla)
Calibration: 'L(1.74, .1,1, 0.025)'
Min Age: 174 Mya
Fossil & Justification: *Protulophila gestroi*. Polish Jura, Lower Jurassic (Słowiński et al., 2020).

Node: Scleractinia
Calibration: 'L(2.37, .1, 1, 0.025)'
Min Age: 237 Mya
Fossil & Justification: Many scleractinian fossils appear abruptly in the fossil record at this time (Stanley Jr., 2003).

Node: Actiniidae
Calibration: 'L(3.09, .1, 0.25, 0.025)'
Min Age: 309 Mya
Fossil & Justification: *Essexella asherae*. Upper Carboniferous, Mazon Creek Formation (Plotnick et al., 2023).

Node: Antipatharia
Calibration: 'L(4.70, .1, 0.25, 0.025)'
Min Age: 470 Mya
Fossil & Justification: *Sinopathes reptans.* Tianjialing, Yichang, Hubei Province, early Arenigian (Baliński et al., 2012)

Node: Octocorallia
Calibration: 'L(1.36, .1, 1, 0.025)'
Min Age: 136 Mya
Fossil & Justification: *Pseudopolytremacis japonica*. Belvédère de Gaud, SW Saint-Remèze, Ardeche, Upper Barremian (Löser & Ferry, 2006).

Node: Cnidaria
Calibration: Skew-normal distribution with 563.7 Mya hard minimum and 609 Mya 5% soft maximum ('SN(5.637, 0.2312, 183.45)').
Minimum Age: 563.7 Mya
Fossil & Justification: *Haootia quadriformis* from the lower Fermeuse Formation, dated to between 563.67–569.01 Mya (Matthews et al., 2021). Has been interpreted as a stem staurozoan, but this is uncertain (Van Iten et al., 2016). We conservatively use this fossil to constrain crown Cnidaria.
Maximum Age: 609 Mya
Justification: Age of the Lantian biota, recently dated to 602 +- 7 Mya (Yang et al., 2022). This formation preserves many exceptional algal macrofossils but no definitive crown metazoans.

Node: Acraspeda

Calibration: 'L(5.419, .1, 0.25, 0.025)'
Min Age: 541.9 Mya
Fossil & Justification: *Paraconularia ediacara* from the Ediacaran Tamengo Formation (Leme et al. 2022), dated to 542.27 ± 0.38 Mya (Parry et al., 2017). Conulariids may have affinity to scyphozoans or staurozoans, but in any case can reliably constrain crown Acraspeda.

Node: Carybdeida
Calibration: 'L(5.05, .1, 0.25, 0.025)'
Min Age: 505 Mya
Fossil & Justification: Specimens UU07021.01 and UU07021.02 from Cartwright et al., 2007. Cambrian Series 3 Marjum Formation (Utah, USA; c. 505 Ma) yields fossils identified as crown cubozoans with likely affinity for Carybdeida (Cartwright et al., 2007; Van Iten et al., 2014).

Node: Chirodropidae
Calibration: 'L(3.09, .1, 0.25, 0.025)'
Min Age: 309 Mya
Fossil & Justification: *Anthracomedusa turnbulli,* Upper Carboniferous, Mazon Creek Formation (Young & Hagadorn 2010).

Node: Scyphozoa+Cubozoa
Calibration: 'L(5.145, .1, 0.25, 0.025)'
Min Age: 514.5 Mya
Fossil & Justification: *Yunnanoascus haikouensis* from the Lower Cambrian Heilinpu Formation (Han et al., 2016), Cambrian Stage 3 (514.5 - 521 Ma). Explicit phylogenetic analyses place as a stem scyphozoan, so we use this fossil to constrain the split between Scyphozoa and Cubozoa.

Node: Rhizostomeae
Calibration: 'L(3.05, .1, 0.25, 0.025)'
Min Age: 305 Mya
Fossil & Justification: *Prothysanostoma eleanorae*, Upper Carboniferous; Cherryvale Formation/Wea Shale, IA. Missourian Stage (Young & Hagadorn 2010).

Node: Coronatae
Calibration: 'L(5.05, .1, 0.25, 0.025)'
Min Age: 505 Mya
Fossil & Justification: Specimen UU07021.05 from Cartwright et al., 2007. Cambrian Series 3 Marjum Formation (Utah, USA; c. 505 Ma) yields fossils identified as crown scyphozoans with likely affinity for Coronatae (Cartwright et al., 2007; Van Iten et al., 2014).

Node: Hydrozoa
Calibration: 'L(5.05, .1, 0.25, 0.025)'
Min Age: 505 Mya

Fossil & Justification: Cartwright et al, 2007; Van Iten et al., 2014: “The oldest known hydrozoans and cubozoans are represented by medusae in the Cambrian (Series 3) Marjum Formation (Utah, USA; *c*. 505 Ma)”

Node: Limnomedusae
Calibration: 'L(2.42, .1, 1, 0.025)'
Min Age: 242 Mya
Fossil & Justification: *Progonionemus vogesiacus*, Lower Triassic; Grès à Voltzia Formation, Middle Triassic (Young & Hagadorn 2010).

Node: Plumularioidea
Calibration: 'L(3.827, .1, 0.25, 0.025)'
Min Age: 382.7 Mya
Fossil & Justification: *Archaeoantennularia byersi* Decker, 1952 and *Plumalina densa* Hall, 1878. Middle Devonian, Sylvania, Michigan, USA and Chemung, Belvidere, New York, USA (Song et al., 2021).

Node: Hydractiniidae
Calibration: 'L(0.66, .1, 1, 0.025)'
Min Age: 66 Mya
Fossil & Justification: *Psammoactinia antarctica* Olivero and Aguirre-Urreta, 1994. Late Cretaceous, Early Maastrichtian, Sanctuary Cliffs, Snow Hill Island, Antarctica (Song et al., 2021).

Node: Porpitidae (stem)
Calibration: 'L(4.211, .1, 0.25, 0.025)'
Min Age: 421.1 Mya
Fossil & Justification: *Pseudodiscophyllum windermerensis*. Late Silurian, Ludlow, Ludfordian, Bannisdale Formation, England (Fryer and Stanley, 2004).

Node: Filifera 1+2 (Proboscidactyla)
Calibration: 'L(1.74, .1,1, 0.025)'
Min Age: 174 Mya
Fossil & Justification: *Protulophila gestroi*. Polish Jura, Lower Jurassic (Słowiński et al., 2020).

Node: Scleractinia
Calibration: 'L(2.37, .1, 1, 0.025)'
Min Age: 237 Mya
Fossil & Justification: Many scleractinian fossils appear abruptly in the fossil record at this time (Stanley Jr., 2003).

Node: Actiniidae
Calibration: 'L(3.09, .1, 0.25, 0.025)'

Min Age: 309 Mya
Fossil & Justification: *Essexella asherae*. Upper Carboniferous, Mazon Creek Formation (Plotnick et al., 2023).

Node: Antipatharia
Calibration: 'L(4.70, .1, 0.25, 0.025)'
Min Age: 470 Mya
Fossil & Justification: *Sinopathes reptans.* Tianjialing, Yichang, Hubei Province, early Arenigian (Baliński et al., 2012)

Node: Octocorallia
Calibration: 'L(1.36, .1, 1, 0.025)'
Min Age: 136 Mya
Fossil & Justification: *Pseudopolytremacis japonica*. Belvédère de Gaud, SW Saint-Remèze, Ardeche, Upper Barremian (Löser & Ferry, 2006).
